# Use of Lean Healthcare to Improve Hospital Throughput and Reduce LOS

**DOI:** 10.1097/pq9.0000000000000473

**Published:** 2021-09-24

**Authors:** Christopher D. Mangum, Rachel L. Andam-Mejia, Leslie R. Hale, Ana Mananquil, Kyle R. Fulcher, Jason L. Hall, Laura Anne C. McDonald, Karl N. Sjogren, Felicita D. Villalon, Ami Mehta, Kyrie Shomaker, Edward A. Johnson, Sandip A. Godambe

**Affiliations:** From the * Department of Quality and Patient Safety, Children’s Hospital of The King’s Daughters; † Department of Patient Care Services, Children’s Hospital of The King’s Daughters, Norfolk, Va.; ‡ Department of Mental Health, Children’s Hospital of The King’s Daughters, Norfolk, Va.; § Department of Supply Chain, Children’s Hospital of The King’s Daughters, Norfolk, Va.; ¶ Department of Pain and Palliative Care, Children’s Hospital of The King’s Daughters, Norfolk, Va.; ∥ Department of Pediatrics, Eastern Virginia Medical School, Norfolk, Va.; ** Children’s Hospital of The King’s Daughters, Norfolk, Va.; †† Department of Emergency Medicine, and Hospital Medicine, Children’s Hospital of The King’s Daughters, Norfolk, Va.

## Abstract

Improving the discharge process is an area of focus throughout healthcare organizations. Capacity constraints, efficiency improvement, patient safety, and quality care are driving forces for many discharge process workgroups.

**Methods:**

Following the Pareto principle, we focused on improving the discharge process on the medical-surgical units that received the most patients admitted from the emergency department. Increased demand for medical-surgical beds, renovations, and diminished bed capacity made it imperative to improve efficiency using quality improvement techniques. A core team of frontline staff decreased the time between computer entry of discharge orders and patient’s departure from the unit to less than 60 minutes, with 80% compliance. The team developed a daily dashboard that detailed the process and outcome measures to create situational awareness and daily visual management. Additional observations of staff workflow uncovered excessive walking for printer use. Printers were placed at the point of use to reduce transport times. Next, using survey results provided by patients on discharge quality, a Treasure Map that aided with teach-back and Team Discharge were implemented to level the staff’s workload. Finally, physicians discharged patients earlier in the day. They standardized their discharge criteria to remove subjectivity from the discharge process and enable better team involvement.

**Results:**

After implementing 4 interventions, the average time between computer entry of discharge orders and patient’s departure from the unit decreased (94.26 versus 65.98 minutes; *P* < 0.001), simultaneously reducing our average length of stay from 5.62 to 4.81 days (*P* < 0.001).

**Conclusions:**

In conclusion, hardwiring proven interventions and complementing them with daily visual management led to significant, sustained results.

## Abstract 2

Supplemental Digital Content is available in the text.

## INTRODUCTION

Improving the discharge process is a common area of focus in healthcare organizations. Capacity constraints, efficiency improvement, patient safety, and quality care are driving forces for many discharge improvement efforts.^1,2^ The discharge of hospitalized pediatric patients may be delayed for various “nonmedical” reasons; such delays impact hospital flow and contribute to hospital crowding.^3^ Data also suggest that inpatient discharge delays affect hospital throughput and contribute to emergency department (ED) crowding.^4^ These delays have also resulted in an unnecessarily increased inpatient length of stay (LOS), diminished hospital revenue, and negative impacts on patient safety and experience.^1^

Our organization faced similar throughput problems, where discharge times from medical-surgical units averaged 94.26 minutes between the computer entry of discharge orders and the patient’s departure from the unit. Our organization’s average LOS (ALOS) was 5.62 days before the initiation of this project, positioning us as the 33rd highest in ALOS among 49 other Pediatric Healthcare Information System (PHIS, Children’s Hospital Association, Kansas City, Mo.) hospitals. During this same time interval (January 2016–September 2016), our hospital was undergoing renovations and had permanently decreased MSU bed space and capacity by 27% to accommodate patients from other hospital areas. This change left the MSU to continue managing 86% of the hospital’s admissions while sustaining an elevated readmission rate. As a result, this quality improvement project aimed to use Lean healthcare tools to generate a culture of change by identifying and reducing waste in the discharge process to improve throughput while reducing the ALOS and readmissions to create more bed capacity without increasing physical bed spaces.

## METHODS

**Fig. 1.**
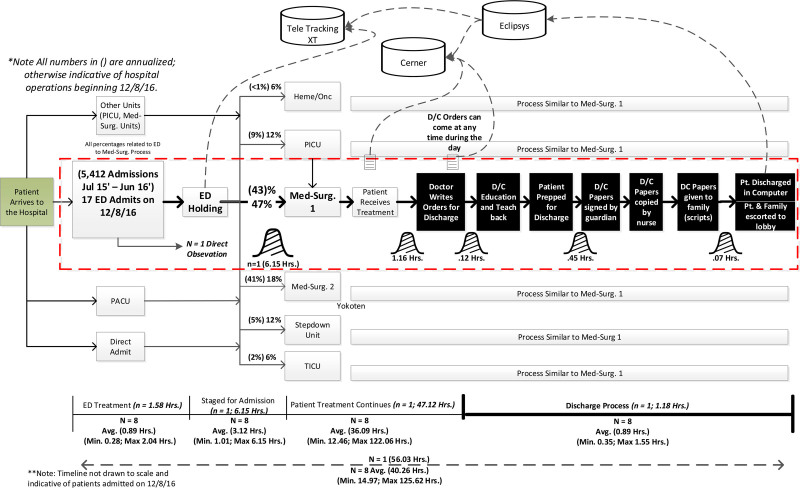
Material information flowchart detailing the throughput or the process of moving patients from the ED throughout the hospital. The material information flow chart also shares a very high level of time-lapse associated with various parts of patient care and the databases the clinicians must interact with to complete this task. The red dotted line identifies the initial area of focus. *All numbers in () are annualized; otherwise, indicative of hospital operations beginning December 8, 2016. **Timeline not drawn to scale and indicative of patients admitted on December 8, 2016.

Our team conducted a quality improvement project with a time-series design at a freestanding children’s hospital on 2 medical-surgical units that initially contained a total of 53 beds. The Pareto analysis was used to select units that admitted and discharged the highest frequency of patients over a year. The scope of this project spanned 4 phases, with each relying on the previous for success. The project team completed observations and time studies to document all process steps in a material information flow chart (Fig. 1) to understand and identify delays in the process and analyze the time associated with various components of the hospitalization process. These efforts allowed the team to visualize the baseline conditions, including the lead time of patients admitted from the ED to one of 2 general care units until discharge from the hospital. The team then used the Pareto principle to prioritize and focus *kaizen* activities on areas with the most recurring delays. (For a definition of Lean/Toyota Production System terms, see **Figure 3, Supplemental Digital Content 3,** which describes table of Lean/Toyota production system terms, http://links.lww.com/PQ9/A311.)

**Fig. 2.**
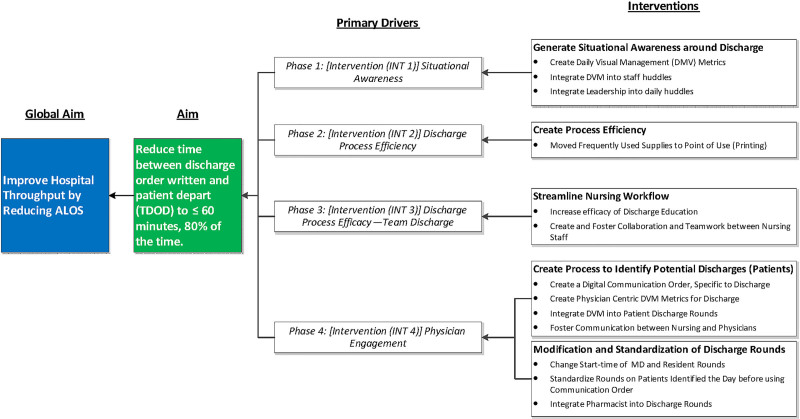
Key driver diagram detailing the scope of each phase of the project and its relevant interventions.

The project team reviewed the lead times of patient throughput using stopwatches during direct observations throughout this project. The team collated the steps recorded during the observations and their respective timestamps to understand all the work that occurred throughout the discharge process. These data were then used to validate the computer-generated timestamps for the larger steps of the discharge process using a measurement system analysis. The area with the most opportunity for improvement was the discharge process. The team then targeted the discharge process to create capacity for patient throughput to reduce the hospital’s ALOS. The project team proceeded with a structured approach to change management within the discharge process as the first phase of change and set a target of generating situational awareness. The second phase of change focused on improving the efficiency of the discharge process through waste reduction. The third phase improved the efficacy of the discharge process. The final phase of improvement targeted changing the time of day attending physicians wrote discharge orders. By moving the time, this order was written earlier to enable the unit to prepare for the increased bed demand from increased ED admissions, which typically increase throughout the day until the late evening. A summary of interventions is displayed in the Key Driver Diagram (Fig. 2) and is further detailed below and in the Results section. This project’s global aim was to reduce the ALOS by reducing the time elapsed between the computer entry of discharge orders and the patient’s departure from the unit to <60 minutes 80% of the time.

### Interventions

#### Phase 1: (Intervention 1) Situational Awareness

**Fig. 3.**
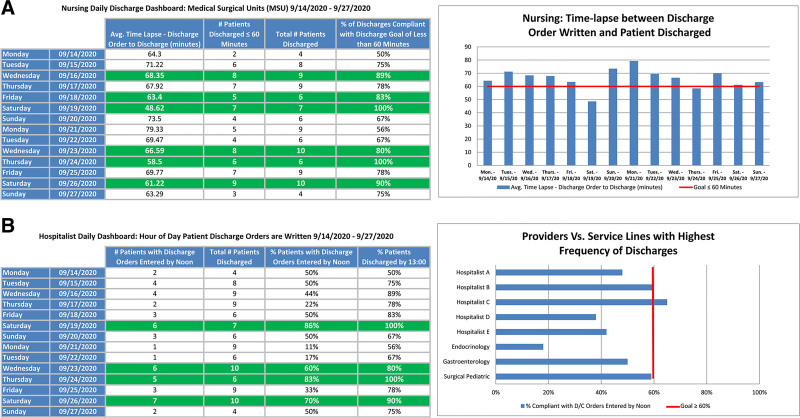
Daily visual management dashboard (discharge process). A and B. Nursing and physician daily visual management detailing the metrics incorporated into daily huddles to drive change transparently. Upon achievement of the goal compliance, the dashboard changes to green to quickly identify success.

The first phase’s primary focus was to generate situational awareness around throughput as a concept, which oriented bedside staff and physicians to discharge patients expeditiously. It allowed room changeover time by the Environmental Service Department and the eventual backfill of these rooms with pending ED admissions. The observed delay with the highest frequency of recurrence was the lack of a sense of urgency. A daily visual management dashboard (Fig. 3A) rallied clinicians around reducing this delay. It contained discharge process-centric key process indicators that became part of the daily shift huddle. During the huddle, nurses discussed the delays for discharge, focusing on problem-solving to mitigate the recurrence of similar themed problems. This daily visual management was created using the PowerInsight data extraction tool (Cerner Corporation, Kansas City, Mo.) and recorded data on all patients discharged from the specified unit within the last 24 hours and displayed them in tabular and graphical format. We then embedded a feedback loop to capture information on all patients not discharged within the 60-minute window of time after computer entry of discharge orders. This feedback loop contained reasons for delays and was managed by the bedside staff completing the discharges.

#### Phase 2: (Intervention 2) Discharge Process Efficiency

The project team continued using the data gathered from the observations and used the Pareto principle to select the next area of focus. During the observations, excessive walking was noted for various materials and supplies frequently used during the discharge process. The team generated Spaghetti diagrams to capture the staff’s walk paths, recognize patterns frequently walked, and identify the materials gathered and used amidst excessive walking. The team used the seven categories of waste to determine what steps in the process were of value from the customer’s (waiting patient’s and family’s) perspective compared to the identified as waste.^5^ The team realized that various supplies were not at the point of use (POU). Single minute exchange of die methodology was paired with the 5S (sort, shine, set in order, standardize, and sustain) methods to place products at the POU.^5–11^ Favorable results occurred on the first MSU. The team scaled this intervention to the second MSU while we continued to analyze the feedback loop in search of additional opportunities for improvement.

#### Phase 3: (Intervention 3) Discharge Process Efficacy—Team Discharge

**Fig. 4.**
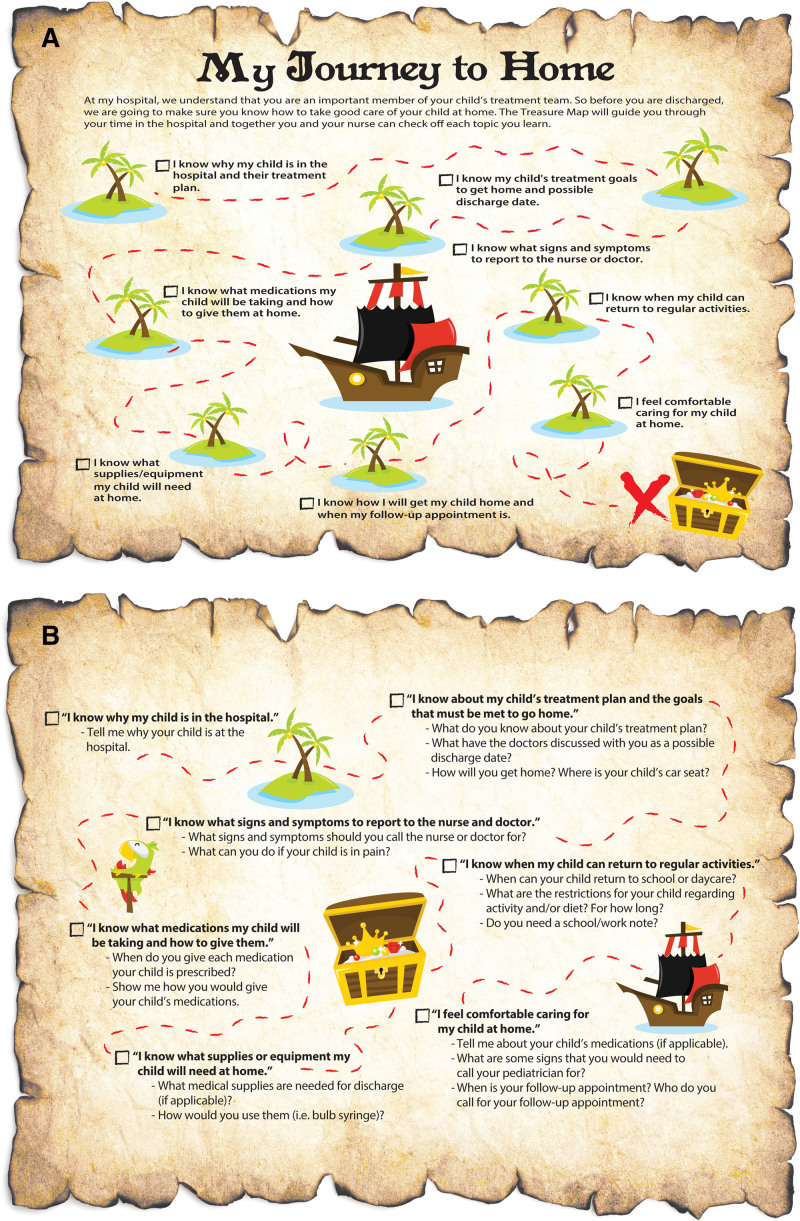
Patient, family and clinician job instruction. A and B. Job instruction used to improve the efficacy of discharge. A, It was used by parents/family to identify where they were in the progression of their child’s hospitalization and to hardwire teach-back methodology by prompting clinicians with questions. Clinicians used (B) to close the communication loop between parents/family and assess the knowledge retained throughout the hospitalization.

After moving supplies to the POU, the next step was to understand the interactions between the bedside staff (nurses, nursing care partners, and unit secretaries) and the patients/families being discharged. Data from the observations revealed the discharge process to be inefficient. Further review of the larger dataset generated from PowerInsight revealed that patients were returning within 7 days for additional treatment for similar diagnoses, which made the team question the efficacy of our discharge process. *Heijunka* (a Toyota Production System principle for leveling the work) was used to improve the efficiency and efficacy of the discharge process.^12^ To complete this, we documented all steps of the discharge process on swim-lane diagrams and the associated time elapsed for each process step. Next, we removed waiting from the process to ensure the continuous flow of value-added work and divided the labor between the unit secretaries, nurses, and nursing care partners. This division of labor allowed them to complete the steps associated with their scope of practice. We then increased the frequency which discharge instruction teaching occurred, from once at discharge to multiple times throughout the hospital stay, and incorporated teach-back to confirm mastery of the instructions.^13–15^ These efforts were completed by incorporating the patient’s parents/guardians into the process with a visual aid (Fig. 4A and B) used as a job instruction (JI).^16^ Figure 4A detailed the 7 learning milestones the patient/family should retain before discharge. At the same time, Figure 4B listed the 7 questions that nursing staff would use to guide the discussion and assess the knowledge retained by the patient/family. The division of labor and JI were coupled together and termed “Team Discharge” (**Figure 1, Supplemental Digital Content 1,** which describes team discharge, http://links.lww.com/PQ9/A309). Once the unit secretary announced “Team Discharge,” this process began, which oriented bedside staff to the next patient discharge location. The bedside staff (nurses and nursing care partners) would note the current time and arrive at the patient’s room to complete their assigned task at the appropriate time as part of teamwork. The team scaled interventions to the second MSU after achieving desired results on the first MSU. We began closely monitoring our 7-day readmissions using PHIS data, whereas the nurses continued to update the feedback loop on patients not discharged within 60 minutes of computer discharge order entry. We analyzed the data and found that over half the delays could be resolved by engaging physicians.

#### Phase 4: (Intervention 4) Physician Engagement

With results from the prior interventions attained and sustained, the team shifted focus to creating capacity and capability to improve overall throughput for patients admitted from the ED while simultaneously reducing ALOS and readmissions. As part of the next intervention, we used reports generated from PowerInsight, manual observations, and data gathered from the nursing feedback loop to better understand the hour of day discharge orders were entered into the computer and the delays associated with entering discharge orders earlier in the day. The team also used Pareto analysis to identify which medical service discharged the highest frequency of patients. The analysis identified the hospitalist team as the group that discharged the most patients, so the team focused on engaging them with discharge process improvement. To accomplish this, we partnered with the organization’s Information Service department to develop a communication order that would invoke the nursing task list, alerting them to prepare the patient/family for an early discharge. The team coupled Spōk technology (Spōk, Springfield, Va.) with the communication order to ensure increased use and ensure it was ordered during evening rounds the day before discharge by sending reminder pages at 14:00 each day. Like nursing, specific to physicians, daily visual management (Fig. 3B) created using PowerInsight visualized the use of the newly designed process. The physician dashboard displayed the percent of discharge orders by noon, compared the progress by medical service, and was posted daily at the POU.

### Data Collection/Measures

The team used PHIS to benchmark our ALOS data against the other 49 participating pediatric hospitals. Next, the team completed manual observations (time and motion studies) to collect data to understand the bedside staff’s work environment, distances walked and the reasons for walking them, as well as the frequency in which they recurred and the quality of the discharge process from the patient’s perspective. The team then completed a measurement system analysis to validate the manual observations by comparing data collected from the Cerner (Kansas City, Mo.) electronic health record. The team used these data to measure and track the overall process, outcomes, and balancing metrics. The PowerInsight data extraction tool (Cerner Corporation) gathered the following data elements for measurement: (1) patient characteristics and demographics; (2) admission and discharge date and times; (3) visit encounter type; (4) discharge order date and times; (5) hospital unit where the discharge occurred; (6) date and times of documented patient education; (7) hospital service of the attending physician writing the discharge order; and (8) return visit information on patients discharged from nursing units included in this quality improvement project.

The team collated all data each month and de-identified it before analysis. This quality improvement project did not meet the criteria for human subject research and, therefore, did not require review and approval by the hospital’s affiliated institutional review board.

### Statistical Analysis

The project team used the data elements mentioned above extracted from the electronic health record and PHIS to create the following variables: (1) time elapsed between the computer entry of discharge orders and the patient’s departure from the unit (process measure 1); (2) compliance with discharging patient’s within 60 minutes of receiving the discharge order (process measure 2); (3) hospital ALOS (outcomes measure); and (4) return visit rate within 7 days of discharge (balancing measure).

The team analyzed all created variables using Minitab Statistical Software v.16 (Minitab, Inc., State College, Pa.). Control charts measured variability throughout the project. The team performed Wilcoxon Rank-Sum Testing (nonparametric) on time-series data to assess improvements and statistical differences in all time-related process and outcomes metrics. We performed a two-sample proportions analysis to test for significance on all attribute data. Additionally, results were considered statistically significant when the alpha statistic (*P* value) was <0.05. Finally, we established a feedback loop to capture and collate reasons for discharge delays when the time elapsed between the computer entry of discharge orders and the patient’s departure from the unit was greater than 60 minutes, which were, in turn, analyzed for frequency of occurrence using Pareto charts.

## RESULTS

**Fig. 5.**
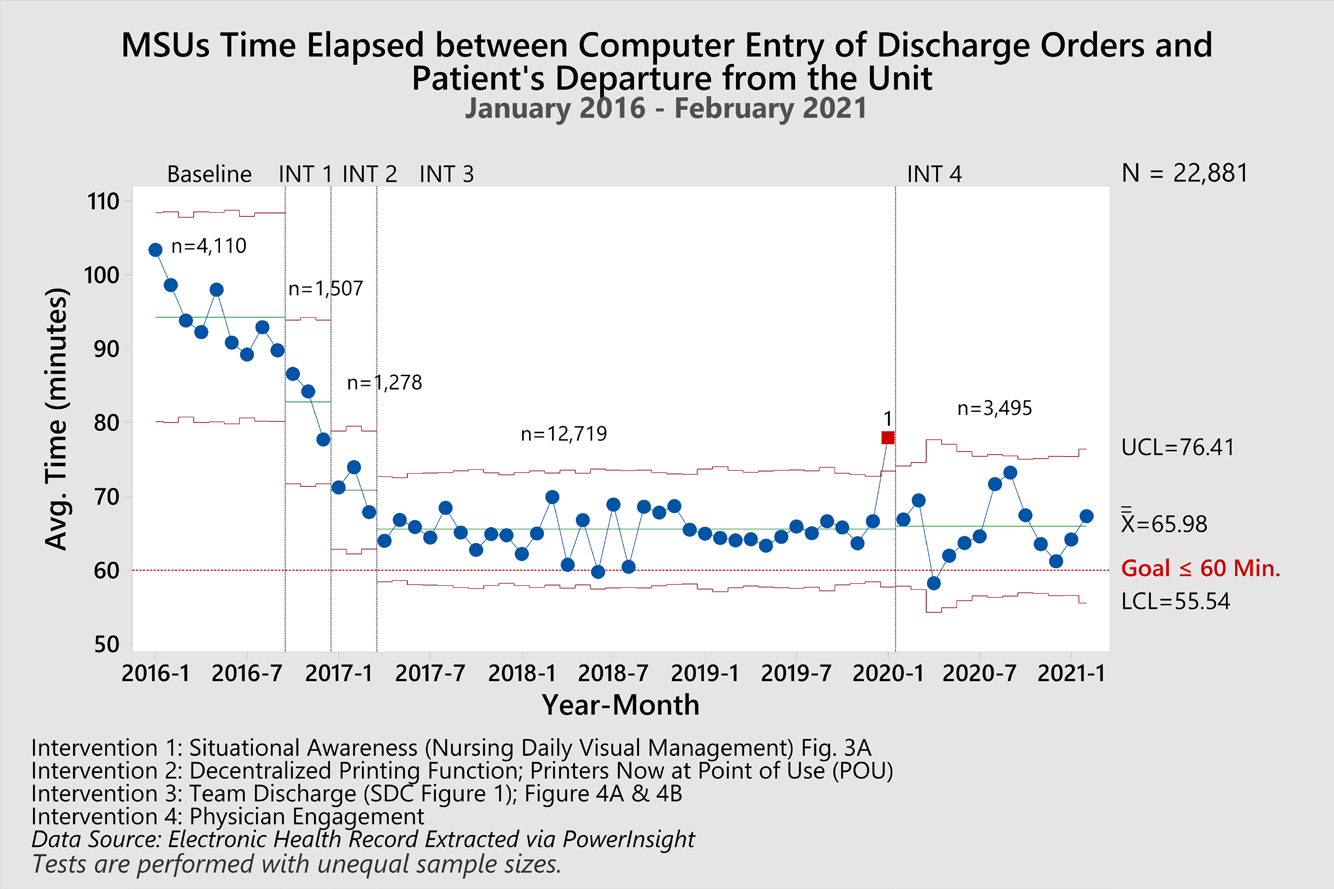
Xbar SPC displaying the time elapsed between computer entry of discharge orders and the patient’s departure from the unit (TDOD). TDOD averaged 94.26 minutes at baseline, and after four interventions, the TDOD averaged 65.98 minutes, a 30.00% decrease, *P* < 0.001. Intervention 1: Situational awareness (nursing daily visual management) Fig. 3A. Intervention 2: Decentralized printing function; printers now at POU. Intervention 3: Team discharge (**Figure 1, Supplemental Digital Content 1**, which describes team discharge, http://links.lww.com/PQ9/A309); Figure 4A and B. Intervention 4: Physician engagement. Data source: PHIS. Tests are performed with unequal sample sizes.

**Fig. 6.**
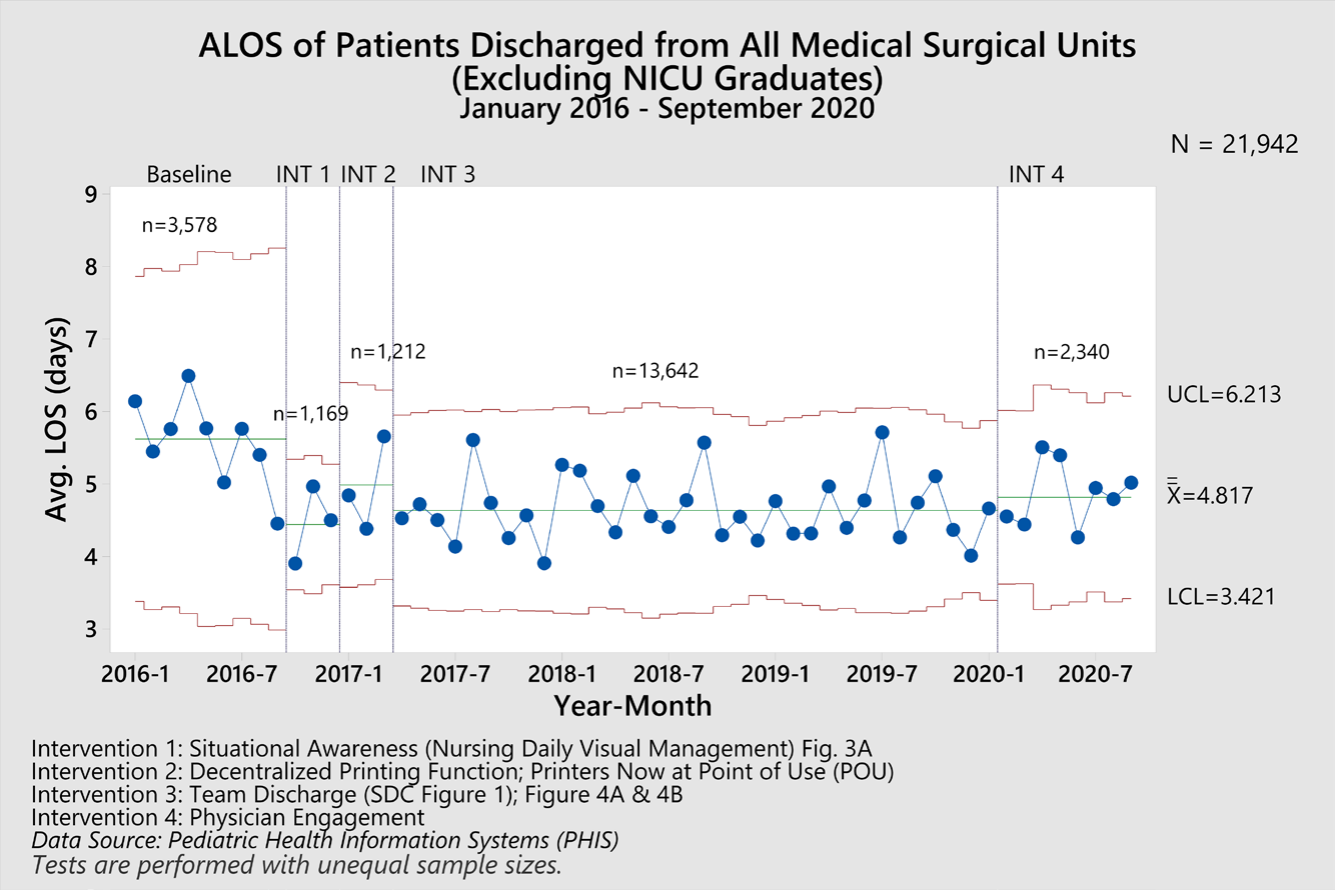
Xbar SPC displaying ALOS across the 4-phased interventions. ALOS averaged 5.62 days at baseline. After all interventions, the ALOS was 4.81 days, a 14.41% decrease, *P* < 0.001. Intervention 1: Situational awareness (nursing daily visual management) Fig. 3A. Intervention 2: Decentralized printing function; printers now at POU. Intervention 3: Team discharge (**Figure 1, Supplemental Digital Content 1**, which describes team discharge, http://links.lww.com/PQ9/A309); Figure 4A and B. Intervention 4: Physician engagement. Data source: PHIS. Tests are performed with unequal sample sizes.

Throughout this study, 22,881 patients were admitted to the MSU. At baseline, patients averaged 94.26 minutes to be discharged from the hospital after a physician had written the discharge order (Fig. 5). After the first intervention (Phase 1: [Intervention 1] Situational Awareness) was implemented, the time elapsed between the computer entry of discharge orders and the patient’s departure from the unit decreased by 12.17% to 82.79 minutes. After the second intervention (Phase 2: [Intervention 2] Discharge Process Efficiency), it decreased by an additional 14.43% to 70.84 minutes. After the subsequent intervention (Phase 3: [Intervention 3] Discharge Process Efficacy—Team Discharge) was implemented, the time elapsed between the computer entry of discharge orders and the patient’s departure from the unit averaged 65.57 minutes, a total of 30.83% reduction from baseline. Finally, after the last intervention (Phase 4: [Intervention 4] Physician Engagement) was implemented, the time elapsed between the computer entry of discharge orders and the patient’s departure from the unit averaged 65.98 minutes. Similarly, the compliance with discharging patients within an hour increased from 42.55% at baseline to 60.15%, a 41.36% change after all interventions. Our ALOS (Fig. 6) decreased by 14.41%, beginning at 5.62 days during the baseline phase of this project to a current value of 4.81 days. This process improvement effort improved our position amongst the other PHIS hospitals, from 33rd highest to 22nd, where lower numbers are better (**Figure 2, Supplemental Digital Content 2,** which PHIS comparison report, http://links.lww.com/PQ9/A310). The team tested all process and outcomes measures for significance compared to these data pre and post all interventions, and all *P* values result less than 0.001. Finally, our readmissions rates (balancing measure) decreased slightly without statistical significance *P* = 0.51, beginning at 3.1% at baseline and 2.9% after all interventions were put into place.

## DISCUSSION

A timely, effective, and efficient discharge process was needed to improve throughput and improve patient outcomes, given the diminished bed capacity from renovations, overcrowding, and increased ALOS. To accomplish this, we took the systematic approach to implement improvements, targeting specific MSU, conducting reiterative plan do study acts, and scaling successful changes to other units, rather than focus change management activities on individual diagnoses and diagnosis related groups as other organizations have.^17,18^ The team chose this method because we realized it was a systems issue impacting all patients, not just patients with specific diagnoses. Taking this approach to solve the problem would have a more significant impact on alleviating common constraints of throughput like patients boarding in the ED and operating theaters, patients waiting in the ED for care and those leaving without being treated, and EDs going on diversion due to lack of capacity to accept ambulance patients.

Although speed was the focus, patient safety was the guiding principle throughout this project. After patients have been deemed ready for discharge, the prolongation of hospital LOS is associated with increased morbidity, mortality, peripheral intravenous infiltrations and extravasations, and hospital-acquired nosocomial infections.^19,20^ There was an expressed focus on mitigating the wait time after the patient was deemed ready for discharge because, during this time, patients on MSU are no longer deemed acute. This designation could potentially cause patient care and coverage to diminish, creating the perfect opportunity for patient harm to occur. We found that one-third of patient adverse events occur during the peak discharge hours, which overlap with the time clinicians are preparing for a shift change, preparing for patient handoff, and during the peak time of receiving new admissions from the ED.

Our organization uses a hybrid methodology for quality improvement to sustain change. It comprises elements from Six Sigma, Lean Healthcare, and the Institute for Healthcare Improvement. This approach focuses on putting the frontline team members in the driver’s seat to foster and implement changes while being coached through a rigorous improvement methodology.

## LIMITATIONS

Data and transparency are needed to drive change. One of the limitations of this project was the inability to acquire real-time data from the medical record on discharge times and the percent of patients with discharge orders written by noon. The team believes this would have given the frontline clinicians the ability to calibrate their discharge processes throughout the day as needed based on immediate feedback. We hypothesize resolving this problem if data is provided to the frontline staff as each patient enters the discharge process.

Case Mix Index (CMI) adjusted LOS is the preferred level of complexity desired when benchmarking against other organizations. However, CMI uses the inpatient status encounter type to calculate CMI. We found that our organization’s disproportionate amount of observation status patients significantly skews the results of the analysis and grossly inflates the results. Therefore, we disregarded CMI in the benchmarking analysis.

## CONCLUSIONS

This quality department-led, frontline team member-driven process improvement project displays how our organization embodies Lean healthcare values. We allowed the team to surface opportunities for improvement associated with a common themed problem the organization was facing, resulting in the improvement efforts mentioned above. Frontline team members have limitless potential when taught the proper improvement tools and empowered to drive change with senior leadership support. Finally, these efforts exemplify how patient-centered our organization’s culture has become by placing parents in the driver’s seat of their child’s care discussion using the discharge JI and team discharge.

## DISCLOSURE

The authors have no financial interest to declare in relation to the content of this article.

## ACKNOWLEDGMENTS

The discharge process improvement team acknowledges the contributions of Bobby Graves and Jamie Bonini of the Toyota Production System Support Center, Inc. (TSSC, Plano, Tex.) and Lyanne Acevedo-Velazquez, Rochelle Davis and Karen Mitchell, and the Information Systems team at The Children’s Hospital of The King’s Daughters (Norfolk, Va.).

## Supplementary Material


