# Supplementary figures and images for: Use of Lean Healthcare to Improve Hospital Throughput and Reduce LOS
Source: Pediatr Qual Saf. 2021 Sep 24;6(5):e473. doi: 10.1097/pq9.0000000000000473 (PMC8476052; doi:10.1097/pq9.0000000000000473)

Team Discharge (Ideal State) v.1

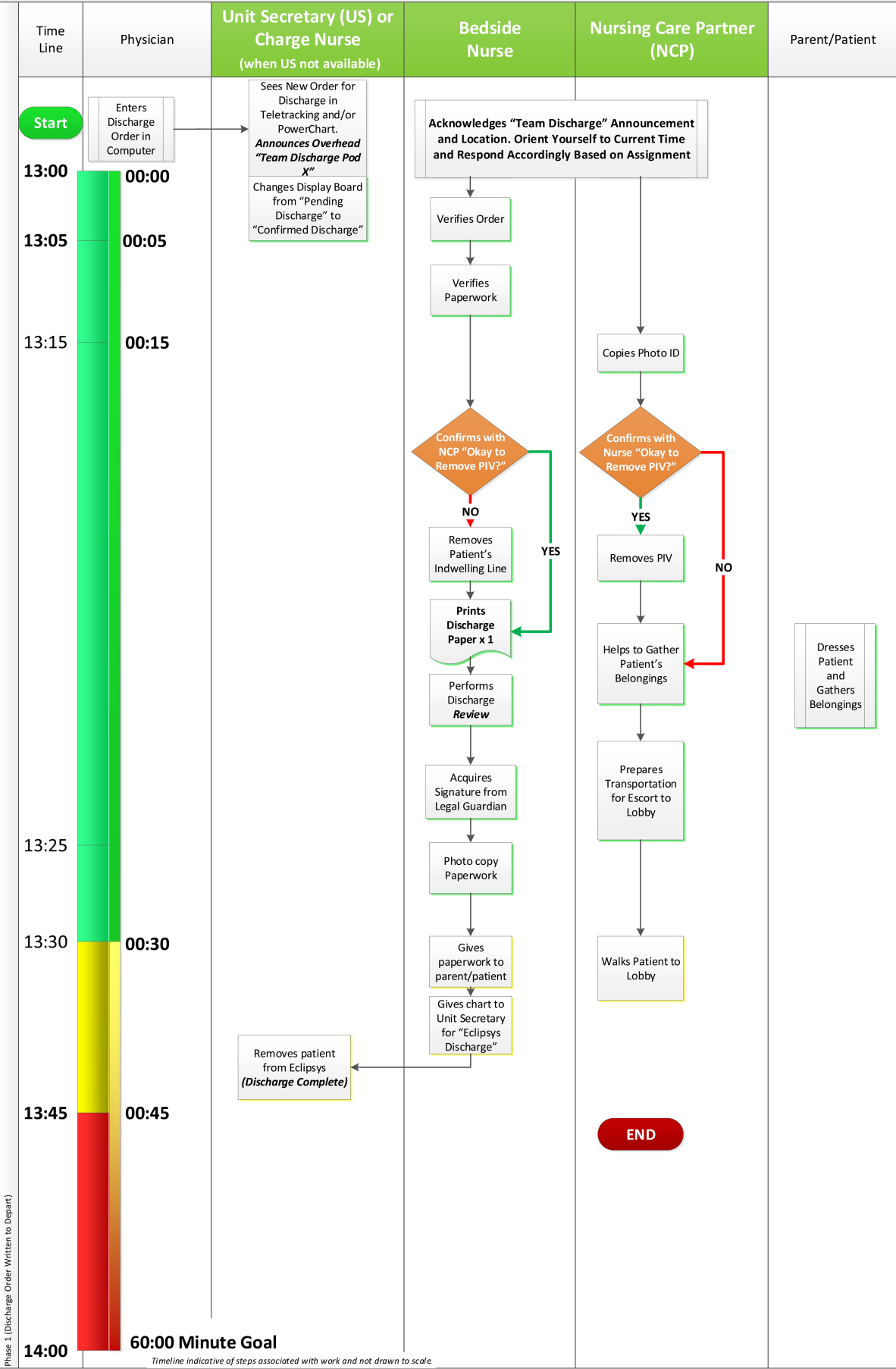

Supplement: Supplementary file 1 [file pqs-6-e473-s001.pdf]
