# Supplementary material for: Use of Lean Healthcare to Improve Hospital Throughput and Reduce LOS
Source: Pediatr Qual Saf. 2021 Sep 24;6(5):e473. doi: 10.1097/pq9.0000000000000473 (PMC8476052; doi:10.1097/pq9.0000000000000473)
Supplement: Supplementary file 2 [file pqs-6-e473-s002.pdf]

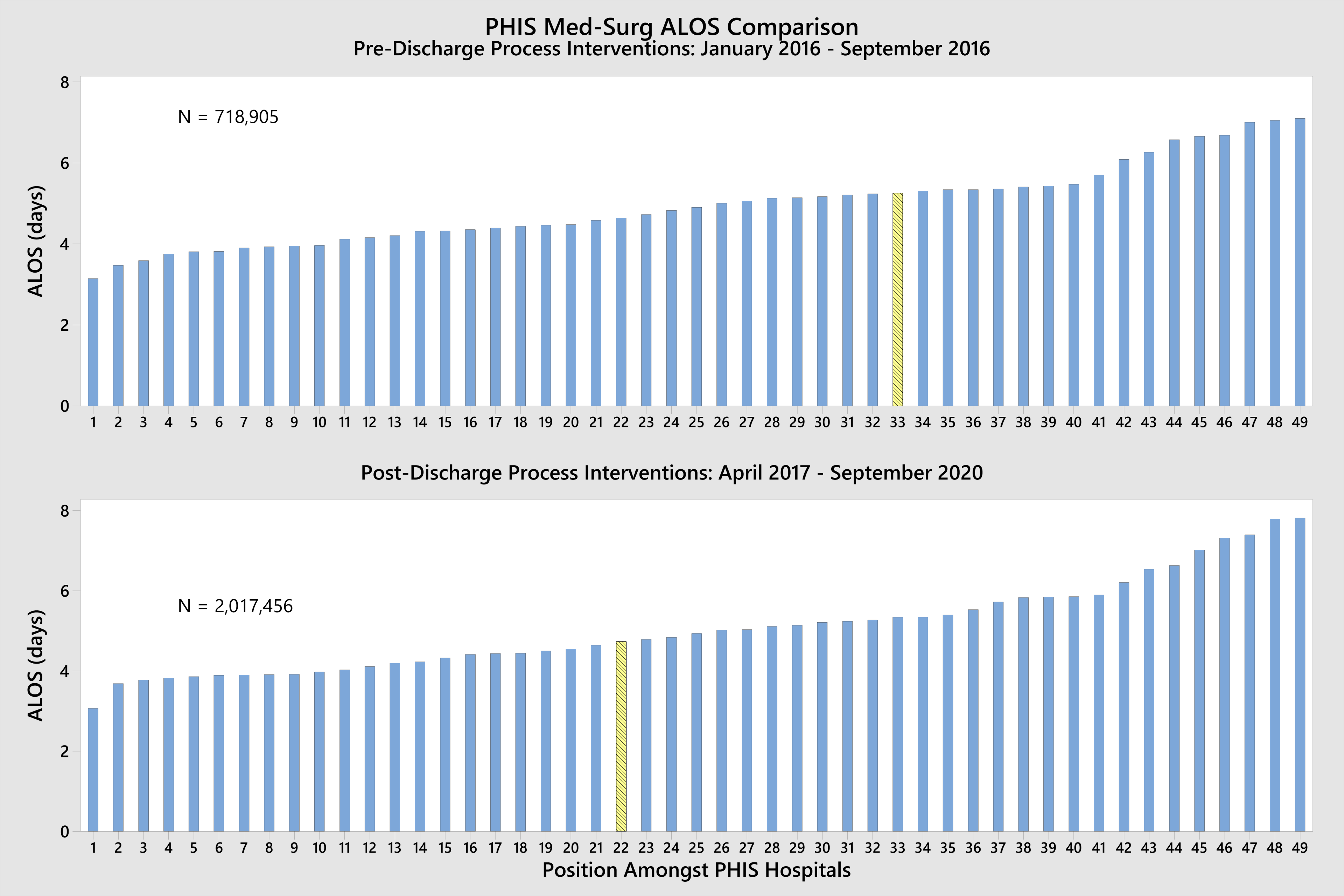
PHIS Med-Surg ALOS Comparison
Pre-Discharge Process Interventions: January 2016 - September 2016
N = 718,905
ALOS (days)
Post-Discharge Process Interventions: April 2017 - September 2020
N = 2,017,456
ALOS (days)
Position Amongst PHIS Hospitals
