# Supplementary material for: Use of Lean Healthcare to Improve Hospital Throughput and Reduce LOS
Source: Pediatr Qual Saf. 2021 Sep 24;6(5):e473. doi: 10.1097/pq9.0000000000000473 (PMC8476052; doi:10.1097/pq9.0000000000000473)
Supplement: Supplementary file 3 [file pqs-6-e473-s003.pdf]

Lean/Toyota Production System Terms	Definitions
1. Material Information Flow Chart (MIFC)	Integration of measurement system and communication with value stream maps
2. Lead Times	Time elapsed between initiation and completion of a production process
3. *Kaizen*	Change for better
4. Changeover Time	Time elapsed between the end of one process and the start of the same process that is expected to yield similar or different products
5. Single Minute Exchange of Die (SMED)	Methodology for reducing changeover time
6. Sort, Shine, Set in Order, Standardize and Sustain (5S)	Methodology used to establish and monitor par level for workspace appropriate material
7. Spaghetti Diagrams	Methodology used to map motion of people and material in a process
8. *Heijunka*	Methodology for leveling work and mitigation of batching by perfectly matching supply with demand of work
